# Usability of a Medication Event Reminder Monitor System (MERM) by Providers and Patients to Improve Adherence in the Management of Tuberculosis

**DOI:** 10.3390/ijerph14101115

**Published:** 2017-09-25

**Authors:** Xiaoqiu Liu, Terrence Blaschke, Bruce Thomas, Sabina De Geest, Shiwen Jiang, Yongxin Gao, Xinxu Li, Elizabeth Whalley Buono, Stacy Buchanan, Zhiying Zhang, Shitong Huan

**Affiliations:** 1 National Center for Tuberculosis Control and Prevention, Chinese Center for Disease Control and Prevention, Beijing 102206, China; leon@chinatb.org (X.L.); jiangsw@chinatb.org (S.J.); gaoyongxin@chinatb.org (Y.G.); lixinxu1978@163.com (X.L.); 2 Department of Medicine, Stanford University, Stanford, CA 94305, USA; 3 The Arcady Group, Richmond, VA 23226, USA; bruce.v.thomas@thearcadygroup.com (B.T.); Elizabeth.whalleybuono@thearcadygroup.com (E.W.B.); stacy.buchanan@thearcadygroup.com (S.B.); 4 Institute of Nursing Science, University of Basel, CH-4056 Basel, Switzerland; sabina.degeest@unibas.ch; 5 PATH China Program, Beijing 100600, China; zzhang@path.org; 6 Bill and Melinda Gates Foundation, Beijing 100027, China; Shitong.Huan@gatesfoundation.org

**Keywords:** adherence, tuberculosis, management, monitoring technology, usability

## Abstract

Poor initiation and implementation and premature discontinuation of anti-tuberculous therapy, all forms of nonadherence, are major reasons for treatment failure, the development of drug-resistant tuberculosis, and transmission to other non-infected individuals. Directly Observed Therapy (DOT) has been the worldwide standard, but implementation of DOT is burdensome for providers and patients, especially in resource-limited settings, where most of the burden of active TB is located. Among the alternatives to DOT is electronic monitoring (EM) of drug dosing histories. Here we report a usability study of a newly-designed, modular electronic monitor product, called the MERM (Medication Event and Reminder Monitor), that is compatible with TB medication formats and supply chains in resource-limited settings. This study, done in a rural setting in China, showed that the use of the MERM for EM of TB medications was associated with a high degree of user performance, acceptability, and satisfaction among both TB patients and medical staff. Based on these data, EM is becoming the standard of care for drug-susceptible TB patients in China and scaled implementations in several other countries with high TB burden have begun. In addition, the MERM is being used in MDR-TB patients and in clinical trials involving patients with TB/HIV and latent TB.

## 1. Introduction

Tuberculosis (TB) is one of the world’s deadliest diseases. Although nearly all cases can be cured, TB remains one of the world’s biggest threats. In 2015, TB killed some 1.8 million people (1.4 million HIV-negative and 0.4 million HIV-positive) [1]. Paradoxically, appropriate treatment cures more than 95 percent of actively-infected patients with drug-sensitive TB [2]. The reasons for treatment failure are multifactorial. Poor medication adherence due to non-initiation of therapy, poor implementation of the treatment regimen, or early discontinuation of treatment are prominent causes [3]. Poorly adherent patients are also at higher risk than adherent patients of developing drug-resistant TB and of passing the disease along to other contacts [1,4]. When effective therapies were initially developed in the late 1940s and early 1950s this resulted in the demise of long-term hospitalization as the standard management of TB and, by 1962, Directly Observed Therapy (DOT) had been implemented in Chennai (formerly Madras), India [5]. The widespread use of DOT was slow until the emergence of multidrug-resistant tuberculosis in New York, and the Advisory Council for the Elimination of Tuberculosis (ACET) made Directly Observed Therapy the standard of care, as a matter of federal policy in 1993 [6]. In a study in New York City comparing DOT with unsupervised therapy, DOT led to significant reductions in the frequency of primary drug resistance, acquired drug resistance, and relapse [7].

In resource-limited settings, however, the challenges of DOT are the labor intensity of DOT, the disruption to the lives and livelihoods of patients, and the costs to the health care system and the patient [8,9]. In the past few years, with the development of a number of new technologies for monitoring and improving adherence, there is a strong interest in evaluating these technologies as a replacement for standard, facility-based DOT, as an adherence aid to the increasingly large number of self-administering patients, and as a mechanism to inform and enable differentiated care. A variety of different innovations have emerged as possible alternatives to DOT. Here we discuss a usability evaluation of a new product, called a “MERM”, an acronym for Medication Event and Reminder Monitor. Usability refers to “the quality of a system with respect to the ease of learning, ease of use and user satisfaction and needs to be tested subjectively, necessitating input from the user’s (i.e., patient or health care provider) perspective” [9]. Usability is typically assessed over the sub-dimensions of user performance, satisfaction, and acceptability [10]. ISO, the International Organization for Standardization, 9241, requires that the context of use for a product be analyzed during product development [11]. Robust usability evaluations are typically conducted throughout a product development lifecycle and strive to answer questions such as: Are users able to carry out their task while expending reasonable resources such as time, cognitive or physical demand? Can the user complete the tasks they are supposed to perform with the product? Is their performance complete and accurate? How do users feel about the performance of the product [12]? Indeed, before introducing new technology such as the MERM in a specific health context, usability testing will allow the identification and resolution of potential issues that might hinder the successful introduction and use of such technology in a research or clinical setting and is therefore considered to be an essential part of studies integrating health technology into patients’ daily lives or health care providers’ clinical practice.

Published literature indicates that electronic monitoring (EM) interventions, including EM-feedback and cognitive-educational interventions, are potentially effective approaches to enhance patient adherence to medications [13,14]. Based upon that knowledge and having a desire to create an electronic monitor compatible for use with blister packaged TB medications, the Chinese National Center for TB control (NCTB) drafted the first Target Product Profile (TPP) that resulted in the original version of the MERM. This earlier version was studied in a cluster-randomized trial (CRT) in China involving 4173 new pulmonary TB patients enrolled across the 36 clusters (ISRCTN46846388) [15]. Despite some equipment and operational issues with this early version related to technical design aspects such as battery life, this study demonstrated the efficacy of a medication monitor to improve medication adherence in TB patients, and showed that reminders from the monitors further improved medication adherence while text messaging reminders did not [15].

## 2. Materials and Methods 

### 2.1. Aims of the Study

To ensure that the new MERM fully addressed the design and usability issues that were identified in the version used in the prior trial by Liu et al. [15] a usability evaluation of the current version of the MERM has been carried out. This study sought to examine the robustness and usability of the new MERM along the subdimensions of user performance, satisfaction, and acceptability to both TB patients and TB providers in a rural setting in China.

The goals of the usability study were:

- The identification of unrecognized defects in the design and function of the MERM prior to scaled deployment [16].
- The very specific identification of patient or provider usability issues, permitting:
  - Targeted instruction, graphics, or FAQs to prevent or resolve such issues.
  - Targeted training to prevent or resolve such issues.
- The assessment of user performance, satisfaction, and acceptability.

This study was small and uncontrolled and not designed to assess the impact of the MERM on the adherence of the participants (patients or providers) involved in the study or to evaluate the potential improved health outcomes of MERM-enabled patient management. These will be objectives of the current large cluster-randomized trial [11]. 

### 2.2. Study Design

This usability evaluation employed a ‘multi-method component design’ that includes a combination of quantitative and qualitative research methods to evaluate three sub-dimensions of usability: user performance, satisfaction, and acceptability [17,18,19].

### 2.3. Setting

The study was carried out in March 2016 by the Chinese Center for Disease Control and Prevention (CDC) at CDC designated TB hospital offices in Luanping and Pingquan counties. Both are Level 2 hospitals in rural counties, serving an average of 13 to 20 new TB patients each month, with approximately 100 TB patients under care at each site. 

### 2.4. Patient and Provider Selection

Patient participants were selected using criterion-related block sampling (e.g., urban versus rural, geographic coverage within China, gender, socio-economic status) to ensure that the usability study participants would closely track the demographics of the planned participant population in the upcoming CRT. Inclusion and exclusion criteria also closely tracked to be used in the upcoming CRT. The initial cohort screened all new TB patients >18 years old who were registered from October 2015 to January 2016 in the two counties. Patients who had been treated for more than five months, who had pleurisy or MDR-TB or who had impaired communication skills were excluded. Phone calls were then made to eligible patients and they were asked whether they were willing to participate in the study when they next came to the TB clinic for follow-up. This process was continued until 15 patients had been recruited at each site. Provider participants consisted of licensed medical providers treating TB population in study region who were willing to implement the MERM and associated ICT System into their practice for a period of three weeks. Ten medical staff (five at each site) were chosen to participate in the study.

### 2.5. Variable and Measurement/Procedures

Prior to the study and before the anti-tuberculous medications were dispensed in the MERM, patients and medical staff at each site received training on the use of the MERM. At the time of inclusion in the study, patient user performance was assessed using a “walk-through and thinking-aloud” method. Patient participants first received detailed, step-by-step instructions as to the critical tasks required for effective use and operation of the MERM monitor. Next, patient participants were then asked to use and to fully operate the MERM monitor, during which they were encouraged to “think aloud”, expressing what they are doing, what they are seeing, what they are thinking, and what they are feeling during such use and operation. At the conclusion of this walk through, thinking aloud session, semi-structured interviews were used to gain further information on specific design or operational issues impacting the practical usability of the MERM monitor by the patients. Following three weeks of “in field” use of the MERM monitor by patients, a semi-structured interview was used to gain information on the satisfaction with and acceptability of the monitors to them. After the interviews the patient participants answered nine questions quantitatively measuring their satisfaction and acceptability, using a five-point Likert scale (1–5), whereby higher scores indicated a more positive experience. Similar approaches were conducted with provider participants to assess the providers’ performance, satisfaction, and acceptability of the MERM monitor. The provider critical tasks were entirely distinct from patient critical tasks, and the usability information reflected these differences. However, the same “walk through, thinking aloud” approach and other qualitative and quantitative methodologies were employed. The providers were also interviewed using a structured questionnaire different than that for the patients. All the interviews and focus group discussions were co-moderated by three professionals not employed at the study sites. Each in-depth interview lasted 20–30 min, and each focus group lasted 40–60 min. 

Table 1 shows the sample size, the dates and the locations of the evaluation in China. 

Table 2 provides available demographic information of the participants. The patients participating in the evaluation ranged uniformly from those under 30 to over 60 years of age.

**Figure 1:**
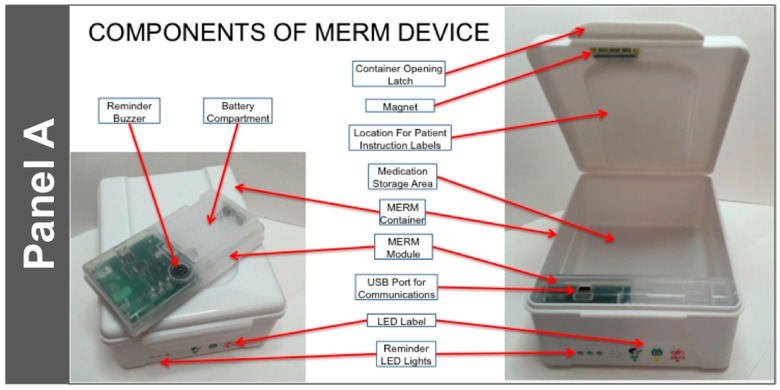

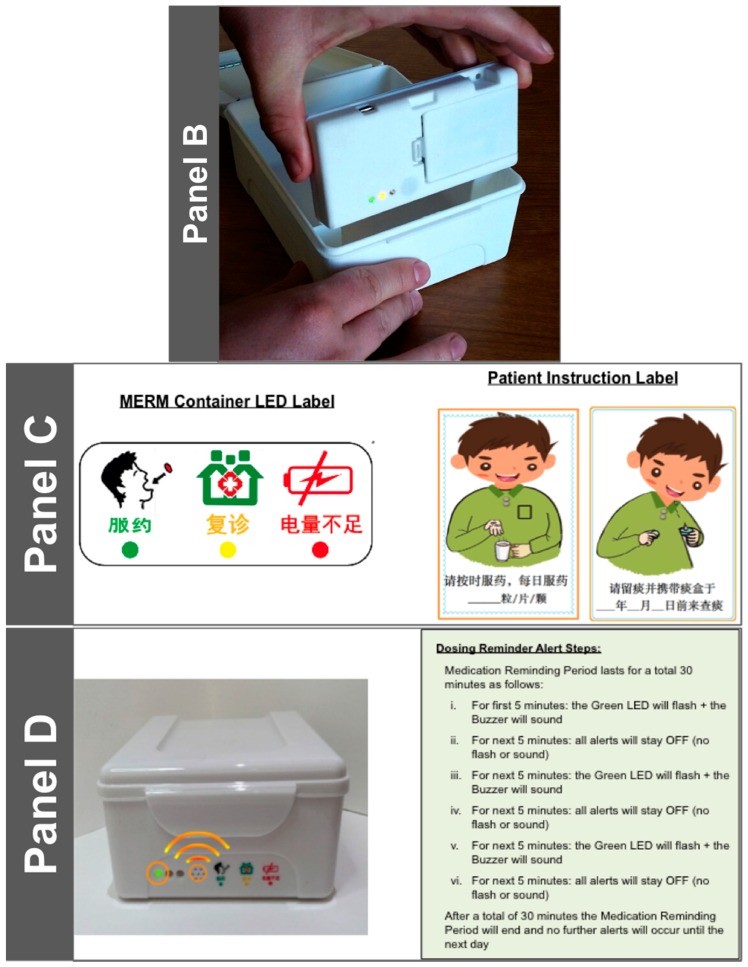
**Panel A** shows the components of the MERM module and its location in the container in which the medication blister cards are stored. As seen in **Panel B**, the MERM module is removable for discrete transportation by the patient and to facilitate data download and battery replacement, if needed. There is ample flat ‘billboard’ space inside the medication container as well as on the top of the container that can be used for patient instruction labels, as shown in **Panel C.** The translation of the Chinese characters in the LED label are: “Take dose now”, “refill medications”, and “low MERM battery”. The left pictogram for the Patient Instruction Label says “please take_X_pills (how many pills) per day” and the right pictogram says “please collect sputum and take the sputum container to the hospital on_(agreed date)”. As also shown in **Panels A**, **B**, and **D**, holes in the container match the locations of the three lights on the MERM (the green dose alert light, the yellow medication refill light, and the red low battery warning light). In addition to the green dose alert light, an audible reminder tone sounds as another dose alert.

Based on subjective feedback gathered from all stakeholders involved in this first trial, an improved monitor, the current version of the MERM (consisting of the MERM electronics module and an accompanying drug container box that stores the medication and securely houses the MERM module), was developed. Pictures of the current MERM and its associated TB medication container are shown in Figure 1.

The salient technological features of the current version of the MERM are as follows:

- It is suitable for use with blister packaged TB medications by TB patients. It can hold one month of DS-TB medication and is highly portable. The electronic module is removable and can, if desired, be transported discretely to the clinic.
- It is highly durable. The container is made of food grade plastic that is resistant to damage from impact. In addition, the MERM has been designed to be resistant to vibration (from transportation or otherwise).
- Data acquisition is simple: It uses a magnetic sensor switch that is triggered upon the opening of the drug container box.
- It has programmable alert mechanisms consisting of:
  - Three LED Lights: Green—Dose Alert; Yellow—Refill; Red—Low Battery
  - An audible tone to indicate that a dose is due.
- At a specific time each day, it produces a ‘heartbeat’ to indicate to the data center that the MERM was functioning that day. The ‘heartbeat’ is downloaded along with the patient dosing data when the patient returns to the clinic for follow up.
- Data transfer is straightforward. A USB port is available to connect to the provider’s data system.
- The power source is simple, and does not require a connection to an electrical outlet or recharging. It utilizes two (2) AA disposable alkaline batteries, and the battery life is greater than 180 days.
- The MERM is affordable: its cost is approximately 9 USD, plus cost of the disposable batteries ($0.50 for the two required). It is highly re-usable, resulting in a lower per-patient cost.

The MERM is being utilized to benefit patients and care providers by helping patients organize their medications, reminding patients of dosing and refill requirements, enabling electronic observation of daily dosing, compiling detailed dosing histories, supporting enhanced counselling of patients, informing and enabling differentiated care. The importance of defining the content of behavior change interventions with precision and specificity [17] will be part of the ongoing CRT. 

Specifically, the use of the MERM in the China CRT and beyond involves the following steps:

1. At treatment initiation, the MERM is registered to the patient and daily dosing refill alerts are set per the medication regimen and patient preference.
2. The MERM is filled with one month of medication and given to the patient.
3. Visual and audible dosing reminders indicate to patients that medication should be taken.
4. Patient opens the MERM and takes medication, which action is captured and stored by the MERM as a proxy for the medication-taking event [20].
5. Patient brings MERM with them to their next provider visit and/or for refill.
6. Care providers connect the MERM via a USB cable to download dosing history and ‘heartbeat’ information, in the process populating the patient-specific adherence dashboard.
7. Care providers print out the patient’s dosing history and use that information to evaluate adherence “patterns”, investigate and understand patient’s specific challenges with medication adherence and counsel the patient about how to recognize and overcome those challenges.
8. The dosing history also drives an algorithm that identifies the patient as high, medium, or low risk, based on the patient’s level and pattern of non-adherence.
9. Refill medications are inserted into the MERM.
10. Daily dosing and refill alerts are adjusted (if required).
11. Patient is sent home again with his/her MERM and medications.

## 3. Results

**Figure 2:**
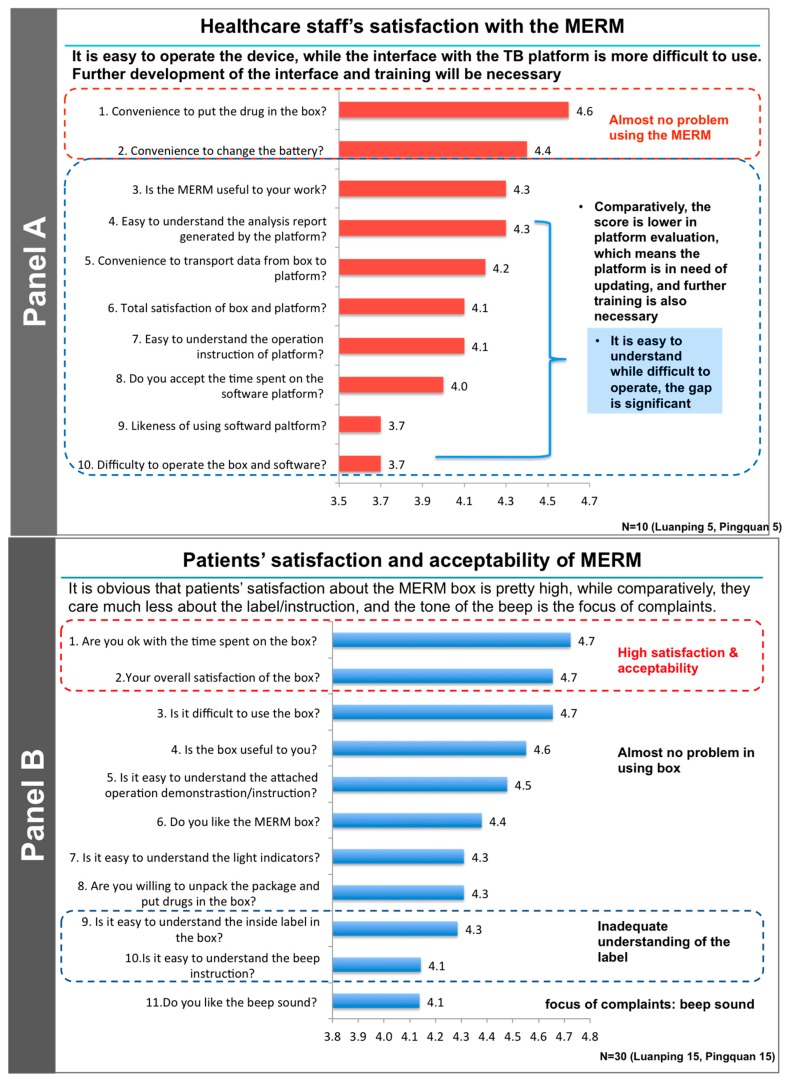
Summary of the structured questionnaires, translated into English, administered to healthcare staff (**Panel A**) and patients (**Panel B**) at both sites. Responses to each question were rated from 1 (low) to 5 (high). Impressions of the investigators are noted to the right of the responses. These results are being used to make modifications to the MERM and with its interface with the China TB software system.

The English translation of the questions can be viewed in Figure 2, which summarizes details of the provider and patient satisfaction and acceptability of the MERM in this usability trial. Activities are currently underway to standardize personalized interventional options for feedback to patients based on the detailed dosing histories available to both providers and patients from the MERM [13,21,22].

After the on-site testing and training of patients with TB prior to the dispensing of their drugs they reported almost no difficulties in using the MERM. They generally indicated that, based on the training, they opened the box only when taking their medications. Having been instructed on how to take the medications, they usually did not spend time re-reading the label that was provided as part of the medication box. At the on-site interview after completion of the three-week study patients reported spending only a very short time getting their medications out of the box every day (usually only 20–30 s from the opening to the closing of the box). The time to use the box differed very little as a function of age. 

The medical staff found that the MERM was easy to use, and that transmission of patient data to the TB data center was convenient. They also noted that the MERM interface was user-friendly and required only a modest amount of on-site training before actual implementation. The medical staff suggested several improvements that were made and will be discussed below. Operationally, the time required by the medical staff to activate and set up the MERM-enabled box was short (under 2.5 min) including installing the batteries, linking the MERM to the desktop computer, setting and adjusting the times of the reminders and putting the drug blister packs into the box. The time required to download and check the data from a MERM from a returning patient was under 30 s.

As outlined above, the identification of changes that could improve the user performance, acceptability, and satisfaction with the MERM was the major goal of this usability study. Many specific comments and recommendations were provided by the medical staff and the patient participants. For example, the medical staff recommended more detailed training, better alignment of the MERM output with the mandatory “TB specific report” required by the CDC, and better formatting of the desktop computer display of the MERM data. Patients suggested more flexibility in the volume and tone of the audible reminder that a dose was due, to distinguish it from similar household sounds, the ability to lock the box to prevent others from using it, and the possibility of battery changes by the patient. Most of these recommendations have been implemented and incorporated in the MERM that is now being employed in a second, ongoing CRT in China (ISRCTN35812455). This is a two-arm, unblinded, pragmatic CRT. Participants in both the intervention and control arms will be given a MERM box and 30 days of medication, which they will be asked to keep in the MERM. Patients in the intervention arm will be required to bring their MERM to each monthly follow-up visit, at which point the doctor or a designee will connect it to the computer and download the data for use in enhanced adherence counselling and to inform and enable differentiated care. In the control arm, the MERM is in silent mode (no audio or visual reminders) and data from the box will be downloaded at the end of treatment. The doctor will not be able to access these adherence data. The unit of randomization will be counties/districts in three provinces in China with the distinction between counties and districts being one of degree of urbanization. The target number of participants in the two trial arms is 3000 patients with drug-sensitive pulmonary TB. Recruitment began in November 2016 and the overall end of the trial, including 18 months of follow up is anticipated to be May of 2019. The primary objective of this latter trial is to evaluate the cost-effectiveness and health outcome impact of patient management supported by the MERM—all as a prelude to large-scale deployment in China.

## 4. Discussion

It is now widely accepted that the challenges of DOT [8,23] preclude its use in resource-limited settings and traditional DOT is practiced only sparsely [24]. A 2017 update of the guidelines for the treatment of drug-susceptible tuberculosis and patient care supports the offering of digital medication monitors to patients with TB [25]. A recent evaluation of the “End TB Strategy” in China, India, and South Africa by nine independent modelling groups examined intervention scenarios that scaled up existing interventions, including adherence support and counseling, and estimated the cost of each scenario [26]. This study concluded that expansion of tuberculosis services would be cost-effective for high-burden countries and could generate substantial health and economic benefits for patients with TB. Relevant to this analysis, our short three-week trial demonstrated that employing the MERM for TB medications was associated with a high degree of user performance, acceptability and satisfaction among both TB patients and medical staff. The patients themselves reported that the MERM was easy to use and improved their adherence and their experience of taking their medications, thereby enhancing their quality of life and that of their families. The medical staff also reported that it reduced the workload and increased their job satisfaction. Currently, the MERM is being implemented in China and India, and is being considered for use in clinical trials and in other chronic diseases such as HIV. There are limitations of this usability study since it was carried out in a specific population and region of China. While the overall impressions of the patients and providers were very positive about the MERM and the recommendations for additional training and modifications were valuable, additional data on its usability will need to be obtained as the MERM is scaled up in other settings in China and other countries.

## 5. Conclusions

The WHO has acknowledged that adherence interventions such as the MERM “significantly improve” treatment outcomes for both DOT and self-administering patients. This usability study has shown that the MERM is highly feasible in resource-limited settings and highly acceptable to patients and providers. This usability study also has led to some recommendations for improvements that have already been implemented as the scale-up of the MERM progresses in both China and India. Importantly, on the strength of this and other trials in China and the evidence therefrom, the WHO, in their recent Guidelines for Treatment of Drug-Susceptible Tuberculosis and Patient Care, 2017 Update, specifically approved digital medication monitors such as the MERM for use by country programs as a suitable “adherence interventions.” Further evaluation of the cost-effectiveness and clinical impact of the MERM is currently underway in a cluster-randomized trial (ISRCTN35812455) in China.

## Figures and Tables

**Table 1:** Evaluation sites and participants at each site.

County of Study in China	Dates of Study	Focus Group Participants		In-Depth Interviews	
		Medical Staff	Patients	Medical Staff	Patients
Luanping	17 March 2016	0	8	5	7
Pingquan	18 and 19 March 2016	5	7	0	8

**Table 2:** Demographics of patient participants.

Age Range (Years)	<30	30–39	40–49	50–60	>60
Number of Participants	5	3	7	8	7

## Supplementary Materials

Supplementary File 1
